# Phase Ib dose-finding trial of lapatinib plus pegylated liposomal doxorubicin in advanced HER2-positive breast cancer

**DOI:** 10.1007/s00280-017-3279-8

**Published:** 2017-03-24

**Authors:** Andrea Rocca, Lorenzo Cecconetto, Alessandro Passardi, Elisabetta Melegari, Daniele Andreis, Manuela Monti, Roberta Maltoni, Samanta Sarti, Elisabetta Pietri, Alessio Schirone, Francesco Fabbri, Caterina Donati, Oriana Nanni, Anna Fedeli, Marina Faedi, Dino Amadori

**Affiliations:** 1 Department of Medical Oncology, Istituto Scientifico Romagnolo per lo Studio e la Cura dei Tumori (IRST) IRCCS, Meldola, Italy; 2 Biostatistics and Clinical Trials Unit, Istituto Scientifico Romagnolo per lo Studio e la Cura dei Tumori (IRST) IRCCS, Meldola, Italy; 3 Biosciences Laboratory, Istituto Scientifico Romagnolo per lo Studio e la Cura dei Tumori (IRST) IRCCS, Meldola, Italy; 4 Oncology Pharmacy Laboratory, Istituto Scientifico Romagnolo per lo Studio e la Cura dei Tumori (IRST) IRCCS, Meldola, Italy

**Keywords:** Breast cancer, HER2, Targeted therapy, Lapatinib, Pegylated liposomal doxorubicin, Phase Ib

## Abstract

**Purpose:**

Combination of anthracyclines with trastuzumab is hampered by cardiotoxicity. Pegylated liposomal doxorubicin and lapatinib could represent a safer alternative to combination therapy.

**Methods:**

In this phase Ib study with 3 + 3 dose escalation design, patients with HER2-positive advanced breast cancer received pegylated liposomal doxorubicin 30 mg/m^2^ intravenously on day 1 plus lapatinib 1250 (level 1) or 1500 (level 2) mg/day orally on days 1–21 of each 21-day cycle. The aims were to establish the maximum tolerated dose at first cycle, and the activity and safety of multiple cycles.

**Results:**

Nine patients out of 11 enrolled were evaluable: 3 at level 1 and 6 at level 2. No dose-limiting toxicities occurred at dose level 1, while 1 (grade 3 diarrhea) occurred at dose level 2, leading to the expansion of this cohort to 6 patients, with no further dose-limiting toxicities. Main grade 1–2 toxicities at first cycle were leucopenia, diarrhea, elevated transaminases, mucositis. Three patients had grade 3 toxicities at subsequent cycles, including colitis, anorexia, stomatitis plus hand-foot syndrome. One partial response, 5 disease stabilizations, and 3 disease progressions were reported.

**Conclusions:**

Combination of pegylated liposomal doxorubicin and lapatinib is feasible and potentially active in pretreated HER2-positive advanced breast cancer patients.

**Trial registration:**

NCT02131506 (ClinicalTrials.gov identifier).

## Introduction

Breast cancer (BC), the most frequent cancer worldwide among women, and one of the main leading causes of cancer death [1], consists of different subtypes according to biology, prognosis, and treatment response [2].

Before the advent of anti-human epidermal growth factor receptor 2 (HER2) therapies, survival of metastatic HER2-positive BC patients was quite poor [3]. The advent of targeted therapies has led to impressive improvements in survival [4]. Nonetheless, long-term outcome data show that only about 12% of patients maintains disease remission after 5 years of first-line trastuzumab-based therapy [5], and almost all patients eventually undergo disease progression and require additional therapies. Although evidence of benefit from HER2-targeted therapies beyond second-line treatment of metastatic BC is not definitive [6], its use is common in clinical practice and is recommended by treatment guidelines [7].

Anthracyclines are among the most active drugs in BC [8], and have been hypothesized [9] to be particularly effective in HER2-positive tumors [10]. Given the cardiac toxicity caused by both drugs [11, 12], the administration of conventional anthracyclines concomitantly with trastuzumab results in unacceptable rates of heart failure, especially in the metastatic setting [4], when long-term therapies are administered. As a consequence, antracyclines remain underused for HER2-positive metastatic BC in clinical practice, with only about 25% of eligible patients treated [13].

Liposomal formulations of doxorubicin appear to maintain similar efficacy with a reduced risk of cardiotoxicity compared to conventional doxorubicin [14]. Pegylated liposomal doxorubicin (PLD) has been approved by the European Medicines Agency for use as monotherapy in patients with metastatic BC in event of increased cardiac risk (PLD summary of product characteristics).

Lapatinib (L), a small molecule, reversible dual tyrosine kinase inhibitor selectively targeting HER2 and epidermal growth factor receptor (EGFR), is indicated for the treatment of adult patients with HER2-positive advanced or metastatic BC in combination with either capecitabine (following progression after therapy including anthracyclines, taxanes, and trastuzumab in the metastatic setting) or trastuzumab (in hormone receptor-negative disease progressing on prior trastuzumab) or with an aromatase inhibitor (in postmenopausal women with hormone receptor-positive disease, not currently intended for chemotherapy) (L summary of product characteristics). L causes low cardiotoxicity, usually consisting in asymptomatic, reversible decreases in left ventricular ejection fraction (LVEF), independently from pretreatment with anthracyclines or trastuzumab [15].

Preclinical work shows an additive cytotoxic effect of the combination of L and PLD in HER2-positive BC cell lines, and a potential synergy in other EGFR-positive/HER2-negative cell lines [16].

We designed a phase Ib clinical trial with the aim to define the maximum tolerated dose (MTD) and the recommended phase-2 dose (RP2D) of this drug combination.

## Materials and methods

### Study design

This was an open label, phase Ib study, with standard 3 + 3 dose escalation design.

The primary objectives were to define MTD, based on the dose-limiting toxicities (DLTs) observed during the first cycle of treatment, and RP2D.

Secondary objectives were to explore the antitumor activity, in terms of objective response rate and clinical benefit, and to assess the safety profile of multiple cycles of therapy, especially cardiac.

Four dose levels were planned: the starting dose level 1, with PLD 30 mg/m^2^ and L 1250 mg/day; dose level 2, with PLD 30 mg/m^2^ and L 1500 mg/day; dose level 3, with PLD 40 mg/m^2^ and L 1500 mg/day; and dose level-1, with PLD 30 mg/m^2^ and L 1000 mg/day.

Cohorts of 3–6 patients were required for treatment at successive dose levels, starting at level 1. If none of the initial 3 patients for each dose level developed a first-course DLT, the following cohort could start 1 dose level higher. If 1 of the initial 3 patients developed a first-course DLT, a maximum of 3 additional patients were to enter at the same level. MTD was defined as the dose level at which ≥33.3% of the patients experienced first-course DLT. If the cohort below MTD, i.e., RP2D, included 3 patients, the cohort was expanded to 6 patients.

DLT was defined as any of the following first-cycle events: grade (G) 4 neutropenia (absolute neutrophil count [ANC] < 500 cells/mm^3^) for ≥5 days, or febrile neutropenia (fever ≥ 38.5 °C with ANC < 1000 cells/mm^3^); G4 thrombocytopenia (platelet count <25,000 cells/mm^3^), or G3 thrombocytopenia associated with a bleeding episode requiring platelet transfusion; *G* ≥ 3 non-hematological toxicity; failure to readminister treatment within 14 days of the planned drug administration due to delayed recovery of treatment-related toxicity to *G* ≤ 1 or baseline.

Toxicity was graded according to the Common Terminology Criteria for Adverse Events (CTCAE) version 3.0.

Treatment was discontinued in case of disease progression, unacceptable toxicity, appearance of intercurrent illnesses or clinical conditions which jeopardized continuation of therapy, or patient decision to withdraw from the study. If interruption of PLD was clinically indicated, patients could continue to receive L monotherapy.

### Study treatment

Each 21-day cycle included L administered orally once daily (1 h before or after breakfast) on days 1–21, and PLD administered intravenously (i.v.) in a 60 minutes’ infusion on day 1 (the first dose was started more slowly to minimize the risk of infusion reactions). Thirty minutes before PLD administration, patients received dexamethasone 8 mg i.v. and ondansetron 8 mg i.v. Anti-emetic medications were prescribed for the following days as needed. Patients were required to comply with the prescribing information for L, including avoidance of inducers and strong inhibitors of CYP3A4; medications for increasing gastric pH may have been taken within 1 h of administration of L, if strictly needed.

### Dose adjustments

Treatment was temporarily withheld in the event of G4 hematological or *G* ≥ 3 non-hematological toxicity or *G* ≥ 2 left ventricular systolic dysfunction (LVSD). Delay of subsequent cycles occurred in case of *G* ≥ 2 hematological on non-hematological toxicity on day 1. Dose reductions were adjusted according to the worst toxicity occurred in the previous cycle, with a reduction of 1 L dose level and 25% of PLD dose in the event of febrile neutropenia, G4 thrombocytopenia or bleeding associated with thrombocytopenia, or *G* ≥ 3 non-hematological toxicity. Definitive treatment interruptions were planned in case of G3 or G4 LVSD, G4 rash manifested as toxic epidermal necrolysis/Stevens-Johnson’s Syndrome, liver toxicity fulfilling Hy’s law (ALT > 3 × ULN and total bilirubin > 2.0 × ULN).

### Safety and tumor assessments

Baseline evaluation included medical history (with concomitant medications) and physical examination, vital parameters and ECOG performance status (PS), cardiologic examination including ECG and echocardiography, laboratory assessments (hematology and blood chemistry, including high-sensitivity troponin T [TnT-hs] and N-terminal pro b-type Natriuretic Peptide [NT-proBNP]), and tumor assessment with CT scan of the thorax and abdomen. Brain CT scan and bone scintigraphy, as well as other imaging studies, were performed if clinically indicated.

Complete blood count and blood chemistry were repeated weekly. Toxicity, physical examination, vital parameters, and PS were assessed before each cycle. Cardiac monitoring included biomarker measurement before (TnT-hs + NT-proBNP) and 24 h after (TnT-hs) PLD administration, as a correlative research evaluation, and ECG + cardiologic exam and echocardiogram (with measurement of LVEF) every 2 cycles of therapy.

Imaging studies for tumor evaluation were performed every third cycle and whenever clinically indicated.

### Patient population

The main inclusion criteria were: histological or cytological diagnosis of BC; locally advanced (stage IIIB–IIIC) inoperable or metastatic (stage IV) disease; HER2 overexpression, defined as +3 staining in immunohistochemistry, or HER2 amplification at fluorescence in situ hybridization (FISH); age ≥18 years; ECOG performance status ≤2; life expectancy ≥12 weeks; disease progression following prior therapy with taxane- and trastuzumab-containing regimens (if not contraindicated); previous cumulative doxorubicin dose ≤240 mg/m^2^; LVEF within the institutional range of normal; adequate bone marrow, liver, and renal functions; and ability to understand and willingness to sign a written informed consent document.

Exclusion criteria included: prior exposure to PLD or L, history of allergic reactions to compounds of similar chemical composition, prior treatment with anthracyclines within 1 year of study entry or prior disease progression while on anthracycline therapy, a pregnant or lactating status, non-compliance with adequate contraceptive measures for patients with reproductive potential, prior or concurrent history of other neoplasms, symptomatic brain metastases, and uncontrolled intercurrent illnesses.

All patients signed a written informed consent. The study was approved by the Scientific and the Ethics Committees of our Institute, and performed in accordance with the Declaration of Helsinki and the Good Clinical Practice guidelines. ClinicalTrials.gov identifier: NCT02131506.

### Statistical analysis

Descriptive statistics are reported for patients’ characteristics and response analysis. The Kaplan–Meier method was used to analyze the time-to-event endpoints. After visually checking homoscedasticity and normality of residual plots, a linear mixed effects model, with random intercepts and slopes, was fitted to evaluate changes of LVEF over time, with time as fixed effect and subjects as random effect. Analyses were done using R (R Core Team 2015) and the R lme4 package.

## Results

Between February 2010 and June 2015, 11 patients were enrolled into the study, 9 of whom were evaluable. A 45-year-old woman with metastatic HER2-positive lobular carcinoma, pretreated with trastuzumab plus pertuzumab plus docetaxel for 2 cycles (in the Cleopatra trial), switched to vinorelbine plus trastuzumab because of an allergic reaction to docetaxel, was considered non-evaluable because of a grade 3 allergic reaction occurring immediately after the beginning of the first PLD infusion. A 65-year-old woman with metastatic HER2-positive lobular carcinoma, pretreated with 2 lines of trastuzumab-based therapy, withdrew her consent before starting therapy.

Characteristics of the 9 evaluable patients are reported in Table 1. Median age was 65 years (range 43–77). All patients had HER2-positive tumors of ductal histology, 7 of whom were hormone receptor positive, and 5 had Ki67 ≥20%. Only 3 patients were pretreated with adjuvant chemotherapy, including anthracyclines in 2 cases, and 2 had adjuvant trastuzumab. All but 1 were pretreated for metastatic disease, including ≥2 lines of trastuzumab-based treatment in most cases. The only patient not pretreated for metastatic disease was a 75-year-old lady who had disease relapse right at the end of adjuvant trastuzumab (prior to T-DM1 registration); she received doxorubicin + cyclophosphamide for 4 cycles and 12 weekly administrations of paclitaxel in the adjuvant setting; at the time of relapse, she refused treatment-induced alopecia, and was not candidate to lapatinib + capecitabine according to the Italian lapatinib indication; therefore, after careful discussion, treatment within the trial appeared a good option and was acceptable for the patient. All had good performance status, although most had ≥2 metastatic sites and visceral involvement.

**Table 1:** Patient and tumor characteristics (for the 9 evaluable patients)

Variable	Median (range), or *n* (%)
Age	65 (43–77)
Performance status (ECOG)	
0	5 (56)
1	4 (44)
Ductal histology	9 (100)
Hormone receptors	
Estrogen	
Positive	7 (78)
Negative	2 (22)
Progesterone	
Positive	5 (56)
Negative	4 (44)
Ki-67	
<20%	4 (44)
≥20%	5 (55)
HER2 status positive	9 (100)
Previous (neo)adjuvant chemotherapy	
With antracyclines	2 (22)
Without antracyclines	1 (11)
Previous (neo)adjuvant trastuzumab	2 (22)
Previous lines of therapy for metastatic breast cancer	
0	1 (11)
1	2 (22)
2	3 (33)
3	3 (33)
Trastuzumab plus taxane^a^	7 (78)
Trastuzumab plus endocrine agent	2 (22)
Trastuzumab plus vinorelbine/capecitabine	4 (44)
T-DM1	2 (22)
Neratinib plus capecitabine	1 (11)
Cyclophosphamide plus docetaxel	1 (11)
Number of metastatic sites	
1	4 (44)
≥2	5 (55)
Sites of metastases	
Soft tissues	2 (22)
Bone	6 (67)
Viscera	6 (67)

^a^In two patients, an endocrine agent was substituted for paclitaxel as maintenance therapy, in combination with trastuzumab, after about 6 months of treatment and an objective response achievement

Of the nine patients, three were enrolled at dose level 1. As none of these patients developed DLT at first cycle, the second cohort started at dose level 2. Among the first three patients of the latter cohort, 1 presented a DLT (G3 diarrhea at first cycle), leading to the expansion of the cohort to 6 patients, with no further occurrence of DLT.

The median number of treatment cycles were 7 for PLD (range 3–13) and 6 for L (range 1–26). The median dose intensity of PLD was 9.4 mg/m^2^/week (range 8.3–10.0). During the whole course of therapy, the median dose intensity of L was 1195 mg/day (range 996–1500) (dose level 1: median 1195, range 996–1225; level 2: median 1220, range 1048–1500).

First cycle toxicities are reported in Table 2. One case of neutropenia and 1 of elevated transaminases represented the only G2 events at first dose level, with G1 leucopenia, nausea, anorexia, and diarrhea as additional side effects. Diarrhea was the predominant adverse event at dose level 2, with 1 case of G3 (DLT) and 2 cases of G1 and G2 each. The only other G2 event was a case of hyperbilirubinemia, which was not associated with elevation of transaminases, and therefore not considered a concern for serious liver injury. Other G1 events included leucopenia, neutropenia, anemia, fatigue, mucositis, epigastric pain.

**Table 2:** First cycle toxicity (*N* = 9 patients)

	Dose level 1				Dose level 2				Overall			
	*n* ^a^				*n* ^a^				*n* ^a^			
	G1	G2	G3	G4	G1	G2	G3	G4	G1	G2	G3	G4
Leucopenia	1	0	0	0	3	0	0	0	4	0	0	0
Neutropenia	1	1	0	0	1	0	0	0	2	1	0	0
Anemia	0	0	0	0	1	0	0	0	1	0	0	0
Fatigue	0	0	0	0	1	0	0	0	1	0	0	0
Nausea	1	0	0	0	0	0	0	0	1	0	0	0
Anorexia	1	0	0	0	0	0	0	0	1	0	0	0
Mucositis	0	0	0	0	2	0	0	0	2	0	0	0
Hyperbilirubinemia	0	0	0	0	0	1	0	0	0	1	0	0
Epigastric pain	0	0	0	0	1	0	0	0	1	0	0	0
Elevated Transaminases	1	1	0	0	1	0	0	0	2	1	0	0
Diarrhea	1	0	0	0	2	2	1	0	3	2	1	0

*G* grade of toxicity

^a^ *n* = number of patients experiencing a given toxicity

Toxicities recorded along the whole treatment period are reported in Table 3. There were no G4 adverse events, and there was only 1 G3 event represented by colitis at dose level 1, and 4 G3 events at dose level 2, represented by diarrhea (already reported above as DLT at first cycle), hand-foot syndrome, mucositis, and anorexia, respectively. The most common G1–2 non-hematological adverse events were hand-foot syndrome, rash, fatigue, mucositis, epigastric pain, hyperbilirubinemia, transaminases elevation, and diarrhea. Hematological adverse events included G1–2 neutropenia in 6 patients, leucopenia in 5 patients, and anemia in 2 patients.

**Table 3:** Overall treatment-related toxicities (*N* = 9 patients)

	Dose level 1				Dose level 2				Overall			
	*n* ^a^				*n* ^a^				*n* ^a^			
	G1	G2	G3	G4	G1	G2	G3	G4	G1	G2	G3	G4
Leucopenia	1	1	0	0	1	2	0	0	2	3	0	0
Neutropenia	1	2	0	0	2	1	0	0	3	3	0	0
Anemia	0	0	0	0	1	1	0	0	1	1	0	0
Hypercholesterolemia	0	0	0	0	1	0	0	0	1	0	0	0
Hand-foot syndrome	0	1	0	0	1	2	1	0	1	3	1	0
Rash	0	1	0	0	1	0	0	0	1	1	0	0
Pneumonia	0	0	0	0	1	0	0	0	1	0	0	0
Fatigue	0	0	0	0	3	2	0	0	3	2	0	0
Nausea	0	0	0	0	1	0	0	0	1	0	0	0
Vomiting	0	1	0	0	0	0	0	0	0	1	0	0
Anorexia	0	0	0	0	0	0	1	0	0	0	1	0
Mucositis	0	2	0	0	3	1	1	0	3	3	1	0
Hyperbilirubinemia	0	0	0	0	1	1	0	0	1	1	0	0
Epigastric pain	1	0	0	0	1	0	0	0	2	0	0	0
Elevated transaminases	2	1	0	0	2	1	0	0	4	2	0	0
Diarrhea	1	0	0	0	2	2	1	0	3	2	1	0
Hypercreatininemia	1	0	0	0	0	0	0	0	1	0	0	0
Hypokalemia	0	0	0	0	1	0	0	0	1	0	0	0
Colitis	0	0	1	0	0	0	0	0	0	0	1	0

*G* grade of toxicity

^a^ *n* = number of patients experiencing a given toxicity (each patient was registered under the maximum grade experienced for each kind of toxicity experienced)

**Fig. 1:**
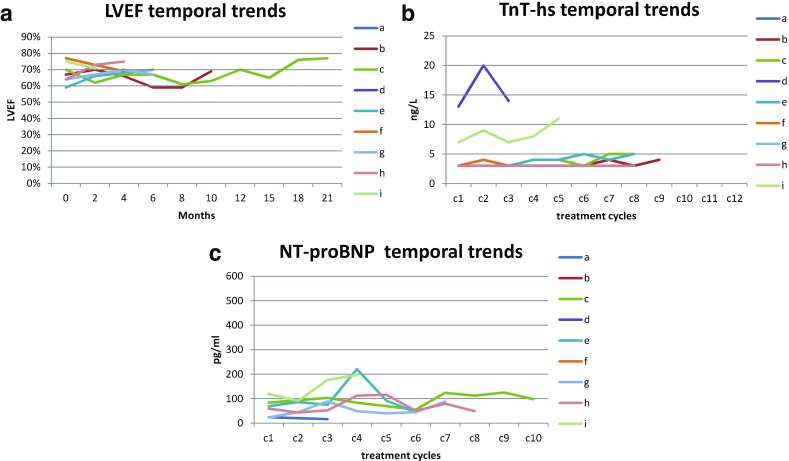
Temporal trends of cardiac parameters and biomarkers: **a** left ventricular ejection fraction (LVEF, normal value ≥55%); **b** high-sensitivity troponin T (TnT-hs, normal value <10 ng/L; rule-in value for myocardial damage >50 ng/L); **c** N-terminal pro b-type Natriuretic Peptide (NT-proBNP, normal value <450 pg/ml)

Regular comprehensive cardiologic evaluations were performed as described above. No cases of LVSD were reported, defined as a LVEF reduction to ≤50 percentage points or a LVEF reduction of >20 percentage points of the baseline value (Fig. 1a), and actually no cases of LVEF reduction ≥10% points of the baseline value occurred. Median baseline LVEF was 67% (range 59–77%, mean 68%) and median LVEF at nadir was 67% (range 59–73%, mean 66%). The analysis of LVEF measures did not show significant changes over time (*p* = 0.26, mixed effects model). Two patients prudently stopped PLD after 13 and 10 cycles, respectively (corresponding to cumulative doses of 390 and 300 mg/m^2^, respectively), but had no significant changes in LVEF. Two patients had temporary increases in TnT-hs levels above the rule-out value for myocardial damage of 10 ng/L, but well below the rule-in value for myocardial damage of 50 ng/L, with values of 20 and 21 ng/L, respectively, which did not increase over time and were considered not clinically significant (Fig. 1b). All 8 patients evaluable for NT-proBNP had levels within normal limits throughout the study period (Fig. 1c).

Temporary interruptions of L were necessary in eight patients (median 6 days, range 2–24), mainly for diarrhea, mucositis, or skin toxicity; delayed administrations of PLD were necessary in five patients (median 1 week, range 1–4 weeks, with subsequent dose reductions in 2 cases), mainly for diarrhea, mucositis, skin toxicity, or leucopenia.

Definitive interruption of either or both study drugs for reasons other than disease progression occurred in 5 patients: one prudently stopped PLD after a cumulative dose of 390 mg/m^2^; one stopped PLD after 9 cycles out of a personal choice; one, pretreated with doxorubicin 240 mg/m^2^, prudently stopped PLD after a cumulative dose of 300 mg/m^2^, and continued with L plus anastrozole outside the study; one stopped L after the first cycle, because of G3 diarrhea (DLT) recurring also after dose reduction to 1000 mg/day, and stopped PLD for medical decision (later switched to maintenance endocrine treatment) after 7 cycles; one stopped L after 2 cycles due to toxicity (G3 anorexia).

Because of the long-term intent of the treatment, and the occurrence of G3 adverse events beyond the first cycle, we decided to discontinue patient enrollment into dose level 3; dose level 2 was considered as the RP2D.

Median follow-up was 22 months. Best responses were: one partial response, 1 stable disease and 1 disease progression at dose level 1; four stable diseases and two disease progressions at dose level 2. Overall, six patients had clinical benefit (objective response or disease stabilization ≥4 months). With a median follow-up of 96 weeks, median time to progression was 23 weeks.

## Discussion

Our study shows that combination of PLD and L is feasible without raising new specific toxicity concerns. In particular, no cardiac adverse events were reported, and an accurate assessment of the circulating cardiac biomarkers TnT-hs and NT-proBNP, along with ultrasonographic studies of the cardiac function, detected no signs of early cardiac damage. No G4 toxicities occurred; the most important G3 events were expected from the use of the two agents, i.e., hand-foot syndrome and mucositis for PLD and diarrhea for L. Liver toxicity was present but ≤G2, and no cases of severe drug-induced liver injury were reported. Furthermore, hematological toxicity was never >G2, ensuring treatment tolerability also in heavily pretreated patients.

The treatment showed hints of activity, with 1 objective response and five disease stabilizations among nine evaluable patients, most of whom were pretreated with ≥2 lines of anti-HER2 therapy for advanced disease.

The pegylated liposomal formulation of doxorubicin favors drug delivery to the tumor, protection from normal organ toxicity, and prolonged half-life [17]. These features contribute to a better therapeutic index than with conventional doxorubicin, as documented by a randomized phase-III trial showing non inferior progression-free survival (PFS) with PLD, with significantly reduced risk of cardiotoxicity [18]. A better cardiac tolerance was confirmed with endomyocardial biopsies in patients treated with high cumulative doses of PLD [19]. Moreover, PLD was proven to be feasible and active in patients pretreated with neo-/adjuvant anthracyclines [20, 21].

L, a reversible dual inhibitor of EGFR and HER2 tyrosine kinases, effectively inhibits the main downstream pathways of these receptors, inhibiting cell proliferation and survival, and inducing the expression of pro-apoptotic molecules [22]. In phase-III randomized trials, L was shown to improve PFS when added to capecitabine or to letrozole in patients with HER2-positive advanced BC [23, 24].

The rate of cardiac events with L, either in monotherapy or in combination with drugs other than anthracyclines, was reported to be 1.6% in a pooled analysis of 3689 patients [15], with mean time to onset of 13 weeks, partial or full recovery in 88% of the cases, and rate of symptomatic congestive heart failure of 0.2%. A rate of 2.2% of cardiac events was observed with L in patients pretreated with conventional anthracyclines [15]. Data on L are consistent with type II cardiotoxicity, characterized by a usually reversible myocyte dysfunction, without necrosis. On the contrary, anthracyclines lead to myocyte damage and necrosis, mainly as a result of topoisomerase-2β inhibition [25]. The reduced myocyte damage occurring with PLD, compared with conventional anthracyclines, and the limited functional damage associated with L, might allow their combined administration. A combination of trastuzumab with non-pegylated liposomal doxorubicin and paclitaxel has been shown to be tolerable, with a rate of grade 3–4 heart failure of 3% and treatment discontinuation due to LVEF decrease in 6% of the patients [26]. The PLD + L combination could be expected to be less cardiotoxic than the above triplet, although its cardiac safety must be evaluated in adequately powered trials.

Both PLD and L are suitable for long-term treatments, which is particularly crucial in the metastatic setting, and is confirmed by the limited toxicity registered in our study. Nonetheless, safety of long-term administration needs be confirmed in larger studies.

Concerning efficacy and potential synergy or additivity, preclinical studies have shown that L blocks efflux pumps such as the breast cancer resistance protein (BCRP/ABCG2), leading to increased intracellular accumulation of chemotherapeutic agents when administered concomitantly [16, 27]. Concomitant treatment of L and PLD showed additive effects in HER2-positive BC cell lines, and a potential synergy in other EGFR-positive, HER2-negative cell lines. Despite some conflicting data [28], this HER2-independent mechanism of action could prove useful especially in heterogeneous tumors, where HER2-positive and HER2-negative subpopulations coexist.

The landscape of pharmacological treatment of advanced HER2-positive breast cancer is rapidly evolving, and the potential collocation of a treatment with capecitabine + lapatinib will depend on new drugs becoming available. Anti-HER2 monoclonal antibodies and tyrosine kinase inhibitors differ in their mechanism of action and resistance [29, 30], and can complement each other when used in combination, concomitantly or in sequence. While other tyrosine kinase inhibitors, such as neratinib, are entering the clinical scenario, L could have a role beyond its combination with capecitabine or letrozole. Because capecitabine can be used in combination with trastuzumab, and could likely be used, in the future, with other drugs like neratinib, a possible use of PLD+ lapatinib is in patients pretreated with capecitabine. Patients intolerant to capecitabine, e.g., for dihydropyrimidine dehydrogenase (DPD) polymorphisms increasing its toxicity, could have the opportunity to receive lapatinib in combination with a non fluoropyrimidine agent.

Both L and PLD were proven to cross the blood–brain barrier, an important aspect in HER2-positive tumors which often lead to brain metastases [31, 32].

The small number of patients enrolled is the main limitation of our study. Slow enrollment was due in part to competing studies on newer anti-HER2 drugs, and in part to difficulties in PLD supply, leading to a recommendation from EMA in September 2011 not to start new treatments with the drug (http://www.ema.europa.eu/docs/en_GB/document_library/Medicine_QA/2011/09/WC500111745.pdf), and solved only in April 2013.

The RP2D emerging from our study is PLD 30 mg/m^2^ every 3 weeks plus L 1500 mg/daily continuously. While PLD has been widely studied (mainly in a first-line setting) at a dose of 50 mg/m^2^/4 weeks, results from phase-II [33] and observational [34] studies showed that lower doses could be equally effective and better tolerated, with a dose intensity of 10 mg/m^2^/week suggested as the best choice [35]. For this reason, and because we were interested in long-term tolerability of the treatment, we decided not to proceed to dose level 3, and to consider dose level 2 as the RP2D.

A phase-II study with the same combination of PLD 40 mg/m^2^/4 weeks plus L 1250 mg daily has been recently reported [36], showing good activity with 54% overall response rate in 24 patients, and good tolerability. Although the intended dose intensity of PLD is the same as in our study (10 mg/m^2^/week), the dose of L is slightly lower. Nonetheless, while our study showed that PLD at 10 mg/m^2^/week can be combined with L at 1500 mg/day in the first cycle, the actual median dose intensity of L during the whole treatment was <1250 mg/day (1220 mg/day), even at dose level 2. Therefore, while selected patients may tolerate L at 1500 mg/day for long periods also in combination with PLD, a dose of 1250 mg/day may be more suitable for most patients. Despite our RP2D for L showed acceptable initial tolerability, dose adjustments may be required to achieve the best therapeutic index.

In conclusion, the limited toxicity of the combination and the hints of activity would support its further development. Given the small and heterogeneous population, larger studies with different design would be needed to draw any conclusion concerning the potential additivity or synergy between the two agents.
